# ﻿Molecular and morphological insights into *Phaeoceros himalayensis* (Notothyladaceae) and related species: evidence for two new species from Thailand

**DOI:** 10.3897/phytokeys.268.172910

**Published:** 2025-12-15

**Authors:** Orawanya Suwanmala, Juan Carlos Villarreal A., Sahut Chantanaorrapint

**Affiliations:** 1 PSU Herbarium, Division of Biological Science, Faculty of Science, Prince of Songkla University, Hat Yai, Songkhla 90110, Thailand Prince of Songkla University Songkhla Thailand; 2 Département de Biologie, Pavillon C.-E. Marchand Université Laval, Québec, Canada Pavillon C.-E. Marchand Université Laval Québec Canada

**Keywords:** Anthocerotophyta, conservation status, Himalayan region, hornworts, spore ornamentation, sporophytes, tubers

## Abstract

The hornwort genus *Phaeoceros* is morphologically diverse, particularly in sporophyte and spore characters. Among its members, *P. himalayensis* and *P. kashyapii*, two previously known species from the Himalayan region, are distinct in their stalked tuber thalli, dark brown sporophytes with valves apically adherent at maturity, and vermiculate spores with or without hump-like projections or verrucae on the distal face. In this study, we combine detailed morphological and molecular evidence to investigate species boundaries within the group of species possessing these traits, comprising *P. himalayensis*, *P. kashyapii*, and related taxa. Within *Phaeoceros*, two fully supported clades were recovered, here recognized as subgenus Phaeoceros and subgenus Himalayanus, **subgen. nov.** Molecular and morphological data also support the recognition of two new species, *P. aequatus* and *P. stenothallus*, both from northern Thailand. The new species are distinguished by unique spore ornamentation together with the production of tubers. These findings support the ancient evolutionary divergence of the Himalayan *Phaeoceros* lineage and underscore the taxonomic significance of spore features and tuber formation. This study adds valuable information to our knowledge of hornwort diversity and evolution, providing a foundation for future systematic and evolutionary studies.

## ﻿Introduction

The genus *Phaeoceros* Prosk. (Notothyladaceae, Anthocerotophyta) is a cosmopolitan group of hornworts that is defined by the absence of internal schizogenous cavities in the thallus, a single chloroplast per cell, the presence of a pyrenoid, antheridia with a non-tiered jacket cell arrangement, the presence of stoma along the sporophyte, and yellow to brownish spores when completely mature (Proskauer 1951; Renzaglia et al. 2009; Villarreal et al. 2010; Suwanmala et al. 2024). The genus represents the largest genus in the family Notothyladaceae, containing some 34 accepted species (Söderström et al. 2016). Of these, *P. himalayensis* (Kashyap) Prosk. and *P. kashyapii* A.K. Asthana & S.C. Srivast. are particularly interesting among the species in this genus, as they have restricted distribution in the Himalayan region and exhibit distinct morphological traits (Asthana and Srivastava 1991; Asthana et al. 2005). The gametophytes of these two species produce long-stalked tubers, and their sporophyte structures differ from those of other *Phaeoceros* species by having yellowish brown to dark brown sporophytes with an adherent tip upon dehiscence and yellowish brown to dark brown mature spores and pseudoelaters. The spores also have irregular verrucae on the distal face. Based on recent molecular studies (Suwanmala et al. 2024; Peñaloza-Bojacá et al. 2025), *P. himalayensis* and *P. kashyapii* are closely related and separated from the major group of *Phaeoceros*.

During the study of *Phaeoceros* in Asia, several morphologically variable populations were discovered that share key gametophytic and sporophytic characters with *P. himalayensis* and *P. kashyapii*, especially their yellowish brown to dark brown sporophytes and adherent sporophyte valves at maturity. The thalli frequently produce the long-stalked tubers. Such morphological variations can lead to misunderstandings of species delimitation. However, differences in spore ornamentation can distinguish these populations and serve as key features for species delimitation, although sometimes it is challenging to ascertain details of spore ornamentation at magnifications available using light microscopy. Additionally, maturity of spores can influence ornamentation patterns, which must be considered during identification. Some recent collections from northern Thailand revealed populations with a novel spore type characterized by rounded spores without protuberances on the distal face, suggesting previously unreported morphological diversity within the genus. Furthermore, some populations exhibited a *Phymatoceros*-like appearance, particularly in gametophytic characters, raising further questions about the boundaries and morphological plasticity within the genus *Phaeoceros*.

Therefore, the aim of this study is to establish a molecular phylogenetic framework to investigate species boundaries within *P. himalayensis* and its related species and to clarify their taxonomic status through an integrative approach combining morphological and molecular evidence. Here, we also describe two new species of hornwort from Thailand based on morphological and molecular evidence.

## ﻿Materials and methods

### ﻿Taxon sampling and morphological investigation

This study is based on recent collections from Thailand as well as herbarium specimens housed in LWG, LWU, QFA, and PSU herbaria. Forty samples of *Phaeoceros* spp. were included in our molecular dataset. *Notothylas breutelii* (Gottsche) Gottsche, *N. javanica* (Sande Lac.) Gottsche, *N. levieri* Schiffn. ex Steph., *N. orbicularis* (Schwein.) Sull., *Notothylas* sp., *Paraphymatoceros diadematus* Hässel, and *Paraphymatoceros* sp. were selected as outgroup taxa. In total, 47 accessions were sampled for phylogenetic analysis, including ten newly generated from recent collections, GenBank sequences retrieved from Suwanmala et al. (2024), and 18 samples from a previously published dataset. The latter were generated using a target enrichment probe technique and published by Breinholt et al. (2021), Bechteler et al. (2023), and Peñaloza-Bojacá et al. (2025). These sequences are available on Dryad (https://doi.org/10.5061/dryad.7pvmcvdqg; Breinholt et al. 2021; https://doi.org/10.5061/dryad.3j9kd51qm; Bechteler et al. 2023) and GitHub (https://github.com/gpenalozabojaca/Hornwort-diversification-.git; Peñaloza-Bojacá et al. 2025). Voucher specimen details, the GenBank accession numbers of newly generated DNA, and the published sequences together with their original sources are provided in Suppl. material 1 (Suppl. material 1: table S1).

Morphological and anatomical characters of *P. himalayensis* and related species were studied using stereo- and compound microscopes. Morphological measurements were taken from fresh collections upon receipt, while herbarium specimens required rehydration before measurement and dissection. Plants were photographed using an Olympus BX51 microscope equipped with a DP74 digital camera and illustrated with the aid of an Olympus drawing tube. In addition, mature sporophytes were selected and air-dried before dissection and deposition of their spores on double-sided adhesive tape attached to aluminum stubs. The stubs with mature spores were coated with a thin layer of gold and examined under an FEI Quanta 400 scanning electron microscope operating at 20 kV.

The preliminary conservation status was evaluated based on the International Union for Conservation of Nature (IUCN) Red List criteria (IUCN 2024), using GeoCAT (Bachman et al. 2011) to calculate the area of occupancy (AOO) and extent of occurrence (EOO).

### ﻿DNA extraction, amplification, and sequencing

Total genomic DNA of silica gel-dried sporophytes was extracted using the E.Z.N.A. Plant DNA kit (Omega Bio-Tek, USA) following the manufacturer’s protocols. We used four molecular markers, comprising one chloroplast marker (*rbc*L) and three hornwort-specific low-copy nuclear markers (L138, L178, and L315) as described in Suwanmala et al. (2024) (Table 1). PCR protocols are listed in Table 2.

**Table 1.** Primer sequences used for PCR amplification and sequencing.

Region	Sequence 5’-3’	Reference
***rbc*L**		
rbcL2_16F	GAGACTAAAGCAGGTGTTGGA	Duff et al. (2004)
rbcL_976R	ACACGAAAGTGAATACCATG	Duff et al. (2004)
**L138**		
Phaeoceros_L138_58F	TTGTCCTGAATTCACGTG GT	Suwanmala et al. (2024)
Phaeoceros_L138_607R	GCTTTGCTAGGGTCTGGTAAG A	Suwanmala et al. (2024)
**L178**		
Phaeoceros_L178_232F	CTCGGGGATGAGCGGGAC	Suwanmala et al. (2024)
Phaeoceros_L178_1088R	GCTTCAAGAGATGGCTCCTT	Suwanmala et al. (2024)
**L315**		
Phaeoceros_L315_676F	GGATTTTGGGGACTTGCACA	Suwanmala et al. (2024)
Phaeoceros_L315_1325R	CTTCTGCCCAACAACAGGAG	Suwanmala et al. (2024)

**Table 2.** PCR conditions for each of the primer sets.

Primer	PCR conditions
*rbc*L, L138, and L315	94 °C for 3 min; 94 °C for 1 min; 55 °C for 30 s, 72 °C for 1 min (35 cycles from 94 °C for 1 min); 72 °C for 10 min.
L178	94 °C for 3 min; 94 °C for 1 min; 58 °C for 30 s, 72 °C for 1 min (35 cycles from 94 °C for 1 min); 72 °C for 10 min.

### ﻿Phylogenetic analysis

Nucleotide sequences were aligned and assembled using Geneious Prime v.2021.1.1(https://www.geneious.com). All forward and reverse sequences were edited and assembled separately using the Geneious alignment tool at a cost matrix of 93% similarity. All sequences of each region, including available sequences from previous publications (see Suppl. material 1: table S1), were aligned separately using the Geneious alignment algorithm with default settings and a cost matrix at 65% similarity. The resulting alignments were adjusted manually and then concatenated. Ambiguous positions were excluded from the alignment, and missing parts of sequences were treated as missing data. The final alignments were uploaded to CIPRES Science Gateway servers (http://www.phylo.org) (Miller et al. 2010) to perform maximum likelihood (ML) and Bayesian inference (BI) analyses.

The ML analysis was conducted using RAxML HPC BlackBox v.8.2 (Stamatakis 2014) with the GTR+I+GAMMA substitution model, following default settings with 1,000 bootstrap replications. Datasets of each single-locus and concatenated sequence were analyzed. A branch with bootstrap support of 70% or higher was considered well supported. The BI analysis was implemented using MrBayes on ACCESS (3.2.7a) (Ronquist et al. 2012) with Markov chain Monte Carlo (MCMC) searches using two simultaneous and independent runs and four chains (one cold and three heated) of 10,000,000 generations. Trees were sampled every 10,000 generations, and the first 10% of sampled trees were discarded as burn-in to ensure convergence of the analyses. Prior to the analyses, the dataset was partitioned by marker into four partitions corresponding to each region (*rbc*L, L138, L178, and L315). The best-fitting model scheme of each region for BI analysis was identified using PartitionFinder 2 (Lanfear et al. 2016). Tracer 1.7.2 (Rambaut et al. 2018) was used to estimate the suitable burn-in and to check the MrBayes output for proper convergence and effective sample sizes (ESS), all of which were >200. Posterior probability (PP) values greater than 0.95 were considered strong support (Erixon et al. 2003). FigTree v.1.4.4 (Rambaut 2017) was used to graph and edit both the maximum likelihood tree and the Bayesian tree.

## ﻿Results

### ﻿Phylogenetic reconstructions

**Figure 1.**
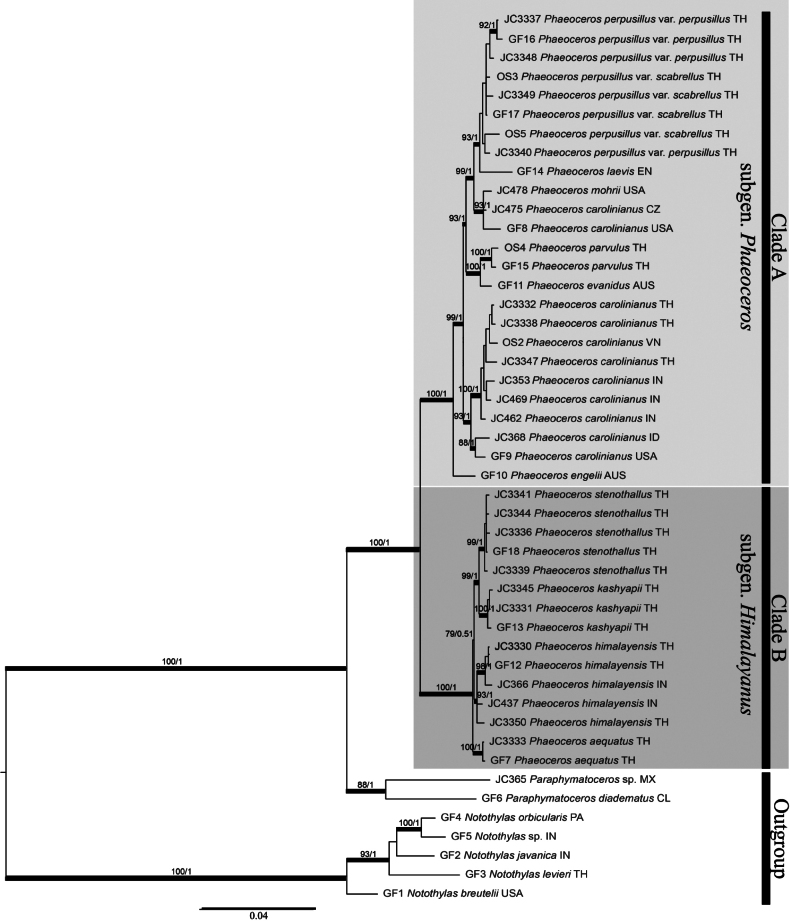
Majority rule consensus tree of phylogenetic relationships of *Phaeoceros* in Asia inferred from *rbc*L, L138, L178, and L315 genes. The two integers above branches represent ML bootstrap support and posterior probability, respectively.

The combined dataset of forty-seven taxa produced a matrix of 2,817 characters, of which 617 were parsimony-informative characters (20.90%). The phylogenetic tree topology obtained from the ML analysis corresponds to that of the BI analysis without any significant conflicts. Therefore, only the topology from the BI consensus tree is shown here along with the bootstrap support values and posterior probabilities (Fig. 1).

The phylogenetic reconstruction shows the monophyletic lineage of the genus *Phaeoceros* with strong support (BS = 100%, PP = 1). In addition, the genus *Paraphymatoceros* was placed as a sister to *Phaeoceros*, with strong support (BS = 88%, PP = 1). There are two main clades within the genus *Phaeoceros*, both strongly supported (BS = 100%, PP = 1). Clade A consists of *P. carolinianus*, *P. engelii*, *P. evanidus*, *P. laevis*, *P. mohrii*, *P. parvulus*, and *P. perpusillus*, with strong support (BS = 100%, PP = 1). Clade B is formed by *P. himalayensis*, *P. kashyapii*, and two undescribed species (*P. aequatus* and *P. stenothallus*) (BS = 100%, PP = 1).

The two undescribed species, *P. aequatus* and *P. stenothallus*, are nested within a clade comprising *P. himalayensis* and *P. kashyapii*, together forming a well-supported monophyletic group. The five accessions of *P. stenothallus* are grouped together with strong support (BS = 99%, PP = 1) and are in a well-supported sister relationship to *P. kashyapii*. They are nested within a clade that includes species producing long-stalked tubers (*P. himalayensis*, *P. kashyapii*, and *P. stenothallus*) (BS = 81%, PP = 0.51). All three assemblages together form a sister group with a well-supported clade of two accessions of *P. aequatus* (BS = 100%, PP = 1).

### ﻿Morphological study

The most informative features of this group are found in the sporophyte. The species have green to yellowish green capsules at a young stage, which become dark brown from the top down and include the base when completely mature. When the sporophytes dehisce, capsules start twisting and form two longitudinal slits along the capsule length. The valves are generally adherent at the tip, rarely opening widely. During the developmental stage, the capsule apex often bends and curves, with a maximum length of up to 15 mm.

Members of subgen. Himalayanus often have irregularly dichotomous branching. Their thallus is more likely to grow scattered as the tips spread out in various directions and typically form dense mats or patches with lingulate or elongated thalli, although they may occasionally grow in obcordate or fan-shaped forms. Except for *P. aequatus*, the latter three species have long-stalked tuber formation. The tubers can grow up to 5 mm long, ending in a globose node or rounded tip, and are sometimes branched. This type of tuber is distributed along the thallus apex, margin, and ventral surface. Due to the unique morphological traits of these four species, we here propose the new subgenus Himalayanus to accommodate them.

Morphological examination and comparison of *P. aequatus* and *P. stenothallus* reveal that they do not belong to any known species. However, the two new species are morphologically aligned with Phaeoceros subgen. Himalayanus, which has been reported from the Himalayan region. The inclusion of *P. aequatus* and *P. stenothallus* in the Himalayan *Phaeoceros* is also supported by molecular evidence, as discussed in the following section. Below, we provide descriptions of four species of the subgenus Himalayanus. A comparison of these species is summarized in Table 3.

**Table 3.** Comparisons of characters between *Phaeoceros aequatus*, *P. himalayensis*, *P. kashyapii*, and *P. stenothallus*.

Characters	*P. aequatus*	*P. himalayensis*	*P. kashyapii*	*P. stenothallus*
Shape of thalli/colonies	lingulate to obovate/irregular patches	lingulate to obovate/irregular patches	lingulate to obovate/irregular patches	lingulate to strap-shaped/irregular patches
Thallus width (mm)	3–7	1–5	2–6	0.8–3
Tuber type	absent	apical, marginal, ventral; long stalk	apical, marginal, ventral; long stalk	apical, marginal, densely ventral; long stalk
Sexuality	monoicous	monoicous	monoicous	dioicous
Number of antheridia per chamber	2–6	2	n/a	2–3
Capsule length (mm)	up to 13	up to 12	up to 15	5–10 (–12)
Distal face of spore	without hump-like structure, finely vermiculate	without hump-like structure, irregular verrucose projections, sometimes with aggregation in center	without hump-like structure, irregular verrucose projections, sometimes with aggregation in center	with hump-like structure, finely vermiculate throughout
Proximal face of spore	finely vermiculate	finely vermiculate with a central depression	finely vermiculate, with minutely papillae on the center of each facet	finely vermiculate
Spore diameter (µm)	30–38	27–35	30–38	29–38

## ﻿Discussion

The resulting topology (Fig. 1) shows that species of *Phaeoceros* fall into two main assemblages (Clade A and Clade B) related to disparate sporophyte and spore morphology. These are Clade A (subgen. Phaeoceros), with yellowish sporophytes having widely opened valves and most species having the spinose spore, and Clade B (subgen. Himalayanus), with yellowish brown to dark brown mature sporophytes having adhering valves and the non-spinose spore. Separation of the clades according to molecular evidence corresponds to different categories of Bharadwaj’s (1981) spore architectures. In his study of Asian hornworts, three types of *Phaeoceros* were grouped based on spore ornamentation, including 1) spinose type, 2) mounded type, and 3) foveate type. Later, the foveate species were transferred to the genus *Phaeomegaceros* (Duff et al. 2007).

Clade A, subgen. Phaeoceros, contains taxa from Asia, America, Australia, and Europe. Species in this clade share similar morphological traits, such as rosette thallus growth, undeveloped tubers (either absent or short-stalked), yellowish capsules with widely spread valves at dehiscence, and spinose spore ornamentation, except for P. perpusillus var. scabrellus spores. Furthermore, all species in this subgenus have a wide distribution range, except *P. perpusillus*. Although this species is abundant where it occurs, its distribution is limited to the northern part of Thailand.

Clade B, subgen. Himalayanus, accommodates all species with irregular patches of thalloid gametophytes, dehiscent sporophytes with an adhering tip of the valves, yellowish brown to dark brown sporophytes at maturity, and distally large verrucae or rounded protuberances on the spores. The strong support across the analysis (BS = 100%, PP = 1) indicates that this group is sister to the other lineage comprising *Phaeoceros* (Clade A). This clade contains four species, *P. aequatus*, *P. himalayensis*, *P. kashyapii*, and *P. stenothallus*, which are restricted to the Himalayan region and northern Thailand. In the field, these species seem to belong to a single variable species, as morphological differences are barely discernible; however, the spores of each differ. The spore ornamentation correlates with each of the four clades in the phylogeny, indicating that morphological and molecular data are congruent.

Within the clade of subgen. Himalayanus, analyses of molecular data from five accessions of *P. stenothallus* support the genetic distinctness of this species, consistent with its morphological characteristics, including a dioicous sexual system, round protuberance on the distal face of the spore surface, narrow thalli with dense tubers, and significantly thick thalli, up to 16 cells thick in cross-section in the middle region. *Phaeoceros stenothallus* is sister to *P. kashyapii*, and together they form a sister clade to *P. himalayensis* with weak support. All three species share the diagnostic characteristic of tuber production. These three lineages together form a sister lineage, with strong support, to *P. aequatus*, which has no tubers on its thalli. Currently, *P. aequatus* is known only from its type locality; however, future investigation may uncover additional populations, which could lead to a better understanding of tuber development in this species.

Based on morphology, Phaeoceros subgen. Himalayanus corresponds to members of *Paraphymatoceros*, the American genus separated from *Phaeoceros* by Hässel de Menéndez (2006). Most species of *Paraphymatoceros* share similar sporophyte characteristics and spore morphology with Phaeoceros subgen. Himalayanus. However, the chloroplasts of *Phaeoceros* have pyrenoids, whereas *Paraphymatoceros* is pyrenoidless. The separation of these two assemblages is also consistent with geographic distribution, as Phaeoceros subgen. Himalayanus occurs in Asia, whereas *Paraphymatoceros* occurs in North and South America.

In the following section, we discuss the distinctive morphological traits of subgen. Himalayanus based on our phylogenetic results.

### ﻿Tubers

Tubers are small outgrowths developed from the thallus and are related to survival mechanisms, as they can help hornworts survive unfavorable conditions. Many hornwort species develop nutrient-filled tubers as perennating structures, allowing them to germinate in the following season (Renzaglia 1978; Renzaglia et al. 2009).

The ability to form tubers has been gained and lost many times during the evolution of hornworts. A sister genus to all *Phaeoceros* species, *Paraphymatoceros*, which has apical flattened and disk-shaped tubers (Hässel de Menéndez 2006; Crandall-Stotler et al. 2008; Renzaglia et al. 2009), suggests that the existence of tubers is the ancestral condition in *Phaeoceros*. *Phaeoceros* clades with tuber-bearing species have higher species numbers than the clade lacking tubers, which comprises only *P. aequatus* and *P. carolinianus*. *Phaeoceros* has been reported to produce short ventral or marginal tubers (Renzaglia et al. 2009; Villarreal et al. 2010), but *P. himalayensis*, *P. kashyapii*, and *P. stenothallus* show tuber morphology similar to the elongated stalked tubers found in *Phymatoceros* (Stotler et al. 2005; Crandall-Stotler et al. 2006). This may be a case of convergent evolution. Abundant tubers with long stalks are morphological traits found in Phaeoceros subgen. Himalayanus, but this trait is likely lost in *P. aequatus*. It is possible that tuber expression may be triggered by environmental factors such as high light intensity and low soil humidity. Individuals of *P. aequatus* at the type locality inhabit a more stable, moist, and shaded environment, which may not induce tuber formation. Thus, having longer tubers possibly enables them to reach deeper soil and access more favorable conditions. Furthermore, the production of long-stalk tubers in *P. himalayensis*, *P. kashyapii*, and *P. stenothallus* might be a response to water stress in somewhat open and dry habitats. Additionally, attenuated thalli or apical tendrils can be found in response to relatively drought-like conditions across different species. Like other hornwort species, the morphology of tubers can vary among *Phaeoceros* species and sometimes can serve as a useful taxonomic characteristic.

### ﻿Sporophyte dehiscence

Most species of *Paraphymatoceros*, a sister genus to all *Phaeoceros* species, share sporophyte morphology similar to Phaeoceros subgen. Himalayanus, suggesting that the adherent tip of the capsule may represent the ancestral condition within the genus. However, further research is needed to establish the actual ancestral state of sporophytes in early *Phaeoceros*. In *Phaeoceros*, capsule dehiscence typically occurs along two longitudinal lines originating near the apex of the sporophyte and proceeding downward (Renzaglia 1978; Renzaglia et al. 2017).

The species of Phaeoceros subgen. Phaeoceros all have yellowish sporophytes splitting into two free valves when they dehisce. These valves tend to twist, especially in the long-sporophyte species such as *P. carolinianus* and *P. laevis*. The occurrence of twisted valves in certain species relates to capsule length and is probably an adaptation to increase spore dispersal. However, within subgen. Himalayanus, sporophytes of the species are characterized by the following features: 1) adherent valves at the apex, 2) a color transition from green at the young stage to yellow at the middle stage and finally dark brown at maturity, and 3) an identical capsule size of approximately 1.2 cm long. The sporophytes are more uniformly short. However, the twisted capsules and unexposed slits suggest that the species is capable of gradual spore dispersal. This strategy might help spore dispersal by water rather than wind, or it may be induced by internal pressure and triggered by environmental changes—particularly the contrast between dry and wet conditions—indicating a more compact and specialized dispersal mechanism.

### ﻿Spore morphology

Morphology of spores has been used as a key feature for species delimitation in many hornwort taxa such as *Anthoceros*, *Dendroceros*, *Notothylas*, and *Phaeoceros* (Hässel de Menéndez 1989, 1990; Chantanaorrapint 2015; Peñaloza-Bojacá et al. 2019; Cargill et al. 2022). However, this structure is conserved in some genera, such as *Nothoceros* (Villarreal et al. 2010). In this study, detailed spore ornamentation is a diagnostic character and useful to define infrageneric taxa within *Phaeoceros*.

Within Notothyladaceae, spores of *Notothylas*, *Paraphymatoceros*, and Phaeoceros subgen. Himalayanus have distally large verrucose or rounded protuberances. Thus, this type of spore is considered the ancestral condition in *Phaeoceros*, and spinose spores are a derived state. However, the transition from spiny to smooth architecture occurs at least once in P. perpusillus var. scabrellus and perhaps in other *Phaeoceros* species from other regions reported by Renzaglia et al. (2009). Despite *Phaeoceros* traditionally being recognized for having spiny spores (Renzaglia 1978), species of subgen. Himalayanus exhibits vermiculate ornamentation with or without hump-like projections or large verrucose coverings on the distal surface and therefore extends the diversity of spore morphologies possessed by *Phaeoceros*.

Although the spore architecture and various features such as the sexual system and tuber morphology of *Phaeoceros stenothallus* resemble those of *Phymatoceros bulbiculosus* (Brot.) Stotler, W.T. Doyle & Crand.-Stotl., molecular phylogeny reconstructions (Fig. 1) unambiguously place *P. stenothallus* within *Phaeoceros*. The size of *Phaeoceros stenothallus* spores ranges from 29–38 µm in diameter, which is smaller than that of *Phymatoceros bulbiculosus* at (49–) 52–64 (–69) µm in diameter (Crandall-Stotler et al. 2006). These two species are morphologically similar and might reflect convergent evolution.

Spore color in *Phaeoceros* ranges from yellow to dark brown. This color is considered a plesiomorphic trait in hornworts and appears in the genus *Phaeoceros* (Renzaglia et al. 2009). Species of subgen. Phaeoceros possess yellow spores, while in subgen. Himalayanus mature spores are yellowish brown to dark brown. The spore color is related to the thickness of the spore walls, and that could contribute to their longevity and drought resistance (Renzaglia 1978; Renzaglia et al. 2009; Peñaloza-Bojacá et al. 2023). Dark spores with thick walls are generally considered more resistant than those with thin walls (Peñaloza-Bojacá et al. 2023). In addition, *Phaeoceros* spores possess oils and starch as storage compounds, which contribute to enhanced longevity and increased resistance to desiccation (Renzaglia et al. 2009).

Spore morphology plays a crucial role in species identification, as even minor differences in spore ornamentation are taxonomically informative. However, certain details of ornamentation, such as the proximal depressions in *P. himalayensis*, can be difficult to observe under a light microscope. Furthermore, spore color may also reflect the stage of maturity. In Asthana and Srivastava (1991), spores of *P. himalayensis* and *P. kashyapii* were reported as yellowish green and pale yellow, respectively. In this study, however, members of subgen. Himalayanus possess darker-colored spores and pseudoelaters compared to subgen. Phaeoceros. The greenish coloration is typically present in immature spores. Spore maturity may also hinder the observation of surface ornamentation. After the capsule splits and releases the spores, or at late maturity, the spore wall architecture in subgen. Himalayanus can be obscured, as the spores are covered by fuscous coating material. This is consistent with observations reported in *Paraphymatoceros* and *Phymatoceros* (Crandall-Stotler et al. 2006, 2008). In contrast, this coating material on mature spores of ripened sporophytes was not found in *P. carolinianus*, *P. laevis*, *P. parvulus*, and *P. perpusillus*.

## ﻿Taxonomic treatment

### ﻿ Phaeoceros

Taxon classificationPlantaeNotothyladalesNotothyladaceae

Prosk., Bull. Torrey Bot. Club 78 (4): 346. 1951.

883B2BF1-F5D4-5B25-8F1D-1199CFFBE06B

#### Type.

*Phaeoceros laevis* (L.) Prosk. (≡ *Anthoceros laevis* L.)

### ﻿Subgenus Phaeoceros

Taxon classificationPlantaeNotothyladalesNotothyladaceae

87F43276-4820-5FE8-995D-72BCEB40771E

#### Diagnosis.

Capsule yellowish to yellowish brown at late maturity, valves two, widely spread at dehiscence. Spores papillate. Tubers sessile or shortly stalked.

#### Included species.

*P. carolinianus* (Michx.) Prosk., *P. engelii* Cargill & Fuhrer, *P. laevis* (L.) Prosk., *P. mohrii* (Austin) Hässel, *P. parvulus* (Schiffn.) J.Haseg., and *P. perpusillus* Chantanaorr.

### ﻿Subgenus Himalayanus

Taxon classificationPlantaeNotothyladalesNotothyladaceae

Suwanmala, J.C.Villarreal & Chantanaorr. subgen. nov.

EF1C6425-4820-5D25-AADA-E953AEF22D98

#### Type.

*Phaeoceros himalayensis* (Kashyap) Prosk. ex Bapna & G.G.Vyas

#### Diagnosis.

Capsule green at early stage, becoming yellowish brown, dark brown to blackish at late maturity, twisted when dry causing 1–2 longitudinally splits along the capsule length, valves usually adherent at tip, rarely widely opened. Tubers absent or present and long-stalked.

#### Included species.

*Phaeoceros aequatus*, *P. himalayensis*, *P. kashyapii*, and *P. stenothallus*.

##### ﻿Key to *Phaeoceros himalayensis* and related species

**Table:** 

1	Plants dioicous; thalli strap-shaped, 0.8–3 mm wide; distal face of the spore with distinct central hump-like structure	***P. stenothallus***
–	Plants monoicous, sometimes strongly protandrous; thalli lingulate to obovate, 1–7 mm wide; distal face of the spore convex without central hump-like structure	**2**
2	Distal face of spores nearly smooth under light microscope; thalli without tubers; pyrenoids stellate	***P. aequatus***
–	Distal face of spores with irregular verrucose projections under light microscope, sometimes with aggregation in center; thalli bearing tubers; pyrenoids smooth	**3**
3	Proximal facets of the spores with a central hollow; thallus lobe 4–12 mm long, 1–5 mm wide	***P. himalayensis***
–	Proximal facets of the spores without a central hollow, but bearing small cluster of minute papillae; thallus lobe 7–20 mm long, 2–6 mm wide	***P. kashyapii***

### ﻿ Phaeoceros aequatus

Taxon classificationPlantaeNotothyladalesNotothyladaceae

Suwanmala & Chantanaorr. sp. nov.

3681BB51-BC2A-5911-B3EC-F991F799BC53

Figs 2, 3, 10A–D

#### Type.

**Thailand** • **Chiang Mai**: Chiang Dao, Pang Wao, 19°24'33.05"N, 098°51'35.46"E, 1,178 m elev., 16 Oct 2020, *S. Chantanaorrapint & O. Suwanmala 4070* (holotype: PSU!; isotype: NICH!, QFA!).

**Figure 2.**
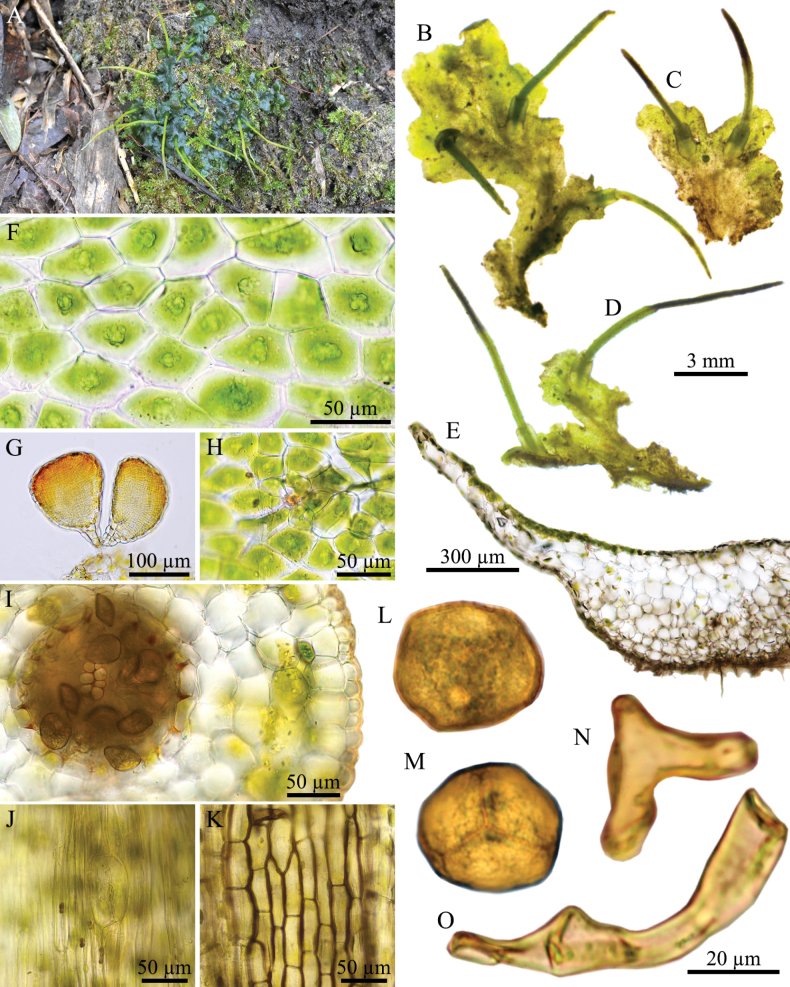
*Phaeoceros aequatus* Suwanmala & Chantanaorr. **A.** Plant in its natural habitat; **B–D.** Gametophytes and sporophytes; **E.** Cross section of thallus; **F.** Dorsal epidermal cells of thallus showing a single chloroplast with pyrenoid per cell; **G.** Antheridia; **H.** Archegonium; **I.** Cross section of capsule; **J.** Epidermal cells of capsule and stoma; **K.** The innermost capsule wall; **L.** Distal view of spore (LM); **M.** Proximal view of spore (LM); **N–O.** Pseudoelaters. Photographed by O. Suwanmala; based on *S. Chantanaorrapint & O. Suwanmala 4070* (**A–K**) and *4092* (**L–O**).

#### Diagnosis.

*Phaeoceros aequatus* is similar to *P. himalayensis* and *P. kashyapii* but differs in the thallus lacking tubers, nearly smooth spores under light microscope (vermiculate under SEM), and the distal face of spores without verrucae.

**Figure 3.**
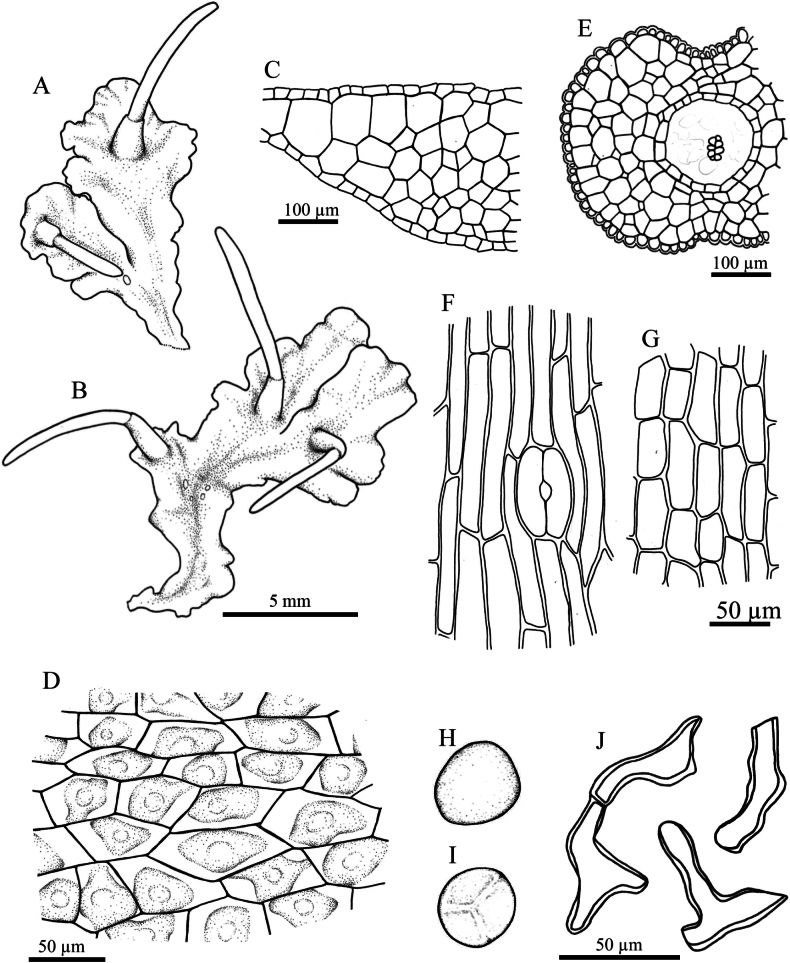
*Phaeoceros aequatus* Suwanmala & Chantanaorr. **A, B.** Gametophytes and sporophytes; **C.** Cross section of thallus; **D.** Dorsal epidermal cells of thallus; **E.** Cross section of capsule; **F.** Epidermal cells of capsule and stoma; **G.** Innermost cells of capsule wall; **H.** Distal view of spore; **I.** Proximal view of spore; **J.** Pseudoelaters. Drawn by O. Suwanmala; based on *S. Chantanaorrapint & O. Suwanmala 4070*.

#### Description.

***Thallus*** green to dark green in fresh material, becoming yellowish green to brown when dry, growing prostrate, moderately adhering to substrate, forming irregular patches or fan-shaped colonies, dichotomous or irregularly branched into several lobes, thallus lobe lingulate to obovate, sometimes fan-shaped, 10–16 mm long, 3–7 mm wide; margins nearly entire to shallowly crenulate, usually flat; apex truncate to round, or shallowly lobulate, rarely attenuate, flat; tubers absent. ***Thallus in cross section*** plano-convex to concave-convex, 5–11 cells thick in the middle region. ***Dorsal epidermal cells*** irregular rectangular to hexagonal, 27–100 × 21–45 µm. ***Chloroplasts*** 1 per cell expanded occupying nearly entire cells, frequently contracted into round shape, pyrenoid stellate. ***Nostoc colonies*** scattered ventrally, appearing as dark dots in old thallus and pale brown near apex. ***Rhizoids*** sparse to densely scattered on ventral surface, smooth in early stage, become pegged at maturity, hyaline to pale brown. ***Sexuality*** monoicous, weakly protandrous. Androecia sparse, slightly raised over the dorsal surface of thallus, usually 2–7 antheridia per chamber; antheridia subglobose to globose, 2–3-tiered stalk with quadriseriate cells, 160–180 × 100–126 µm. Archegonia embedded in thallus, connected to the upper surface, sparse, randomly scattered nearly thallus apex. ***Involucres*** erect, conical-cylindrical, (1.2–)1.5–3 mm long, 3–5 cells thick, mouth smooth, rarely shallowly sinuate. ***Sporophytes*** frequent, capsules erect, sometimes inclined, cylindrical, up to 13 mm long; epidermal cells of capsule elongate-rectangular, 145–243 × 15–25 µm; stomata 77–88 × 45–50 µm, surrounded by 5–6 epidermal cells; assimilative layer 3–5 cells thick in cross section; the innermost cells of capsule elongate-rectangular to hexagonal, 40–103 × 17–33 µm, pale brown to dark brown; columella consisting of 4–6 cells in cross section, reddish brown to dark brown. ***Spores*** yellowish brown to dark brown, rounded-triangular in polar view, 30–38 µm in equatorial diameter, nearly smooth under light microscope (LM); distal face convex with finely vermiculate ornamentation, without hump-like projection; proximal face with thin triradiate mark, bordered by vermiculate strip on each side of trilete mark, each facet covered with fine vermiculate pattern. ***Pseudoelaters*** pale to dark brown at maturity, thick-walled, occasionally branched, 1–3 cells long; pseudoelaters cells rectangular, without helicoidal band.

#### Etymology.

The epithet “*aequatus*” refers to smooth distal surface of spore, as observed under light microscope.

#### Distribution, habitat, and ecology.

*Phaeoceros aequatus* is currently known only from the type locality at Chiang Dao Wildlife Sanctuary, Chiang Mai province, and may represent an endemic species of northern Thailand. It occurs on soil on the edge of the mixed bamboo-pine-oak deciduous forests at elevations of 1100–1200 m. It grows associated with other bryophytes such as *Entodon macropodus* (Hedw.) Müll.Hal., *Fissidens* spp., *Notothylas javanica* (Sande Lac.) Gottsche, and *Phaeoceros carolinianus* (Michx.) Prosk.

#### Conservation status.

The type locality of *Phaeoceros aequatus* is within the Chiang Dao Wildlife Sanctuary, a protected area. Only two small populations of the species have been found along the walking trail, occupying less than a quarter of a square metre. These populations have persisted over six years (2016–2021) of observations. The site remains susceptible to human activities and might be destroyed by fire. However, as suitable habitats appear to occur in the surrounding landscapes and additional survey effort is needed, we propose to treat *P. aequatus* as Data Deficient (DD) until further information becomes available.

#### Specimens examined.

**Thailand** • **Chiang Mai**: Chiang Dao, beside the road from Chiang Dao to Muang Kong, 19°24'47.41"N, 098°49'58.88"E, 1,118 m elev., 28 Oct 2018, *S. Chantanaorrapint & O. Suwanmala 3447* (PSU); • Pang Wao, 19°24'33.05"N, 098°51'35.46"E, 1,178 m elev., 13 Nov 2016, *S. Chantanaorrapint & O. Suwanmala 716* (PSU), 16 Oct 2020, *S. Chantanaorrapint & O. Suwanmala 4070* (PSU), 3 Oct 2021, *S. Chantanaorrapint & O. Suwanmala 4092* (PSU).

#### Taxonomic notes.

The distinctive features of *P. aequatus* are its monoicous sexuality, absence of thallus tubers, nearly smooth spores under a light microscope, vermiculate spores under SEM, and absence of hump-like projections on the distal surface of the spore. *Phaeoceros aequatus* could be confused with *P. himalayensis* and *P. kashyapii*, as they share similar characters of gametophytes and sporophytes, such as ligulate to obovate thallus lobes, monoicous sexual condition, cylindrical capsules with yellowish brown to dark brown color at maturity, adherent tips of capsule valves, and yellowish brown to dark brown spores and pseudoelaters. However, *P. himalayensis* and *P. kashyapii* differ by having irregular verrucose ornamentation on the distal face of the spores that is visible with a light microscope and by producing thallus tubers.

In spore ornamentation, *P. aequatus* is similar to P. perpusillus Chantanaorr. var. scabrellus Suwanmala & Chantanaorr., which also has nearly smooth spores under a light microscope. However, *P. aequatus* differs in having yellowish brown to dark brown spores and longer pseudoelater cells that are more than 1.5 times the spore diameter. In contrast, P. perpusillus var. scabrellus has yellow spores and shorter pseudoelater cells that are as long as the spore diameter.

Similarly, the spores of *Phaeoceros dendroceroides* (Steph.) Hässel resemble those of *P. aequatus*, as both appear nearly smooth under a light microscope. However, *P. dendroceroides* can be distinguished by its broad thallus (6–10 mm wide), which is more consistent with members of the genus *Phaeomegaceros* (Villarreal pers. comm.).

### ﻿ Phaeoceros himalayensis

Taxon classificationPlantaeNotothyladalesNotothyladaceae

(Kashyap) Prosk. ex Bapna & G.G. Vyas, J. Hattori Bot. Lab. 25: 88. 1962.

6C3874E8-CBD1-5226-BAB1-5B44BA66E01B

Figs 4, 5, 10E, F

≡ Anthoceros himalayensis Kashyap, New Phytol. 14(1): 8. 1915.

#### Type.

**India** • **Western Himalayas**: Mussoorie, Kashyap’s illustration (lectotype: Illustration in New Phytologist 14: 8. fig. 4.4. 1915; designated by Chantanaorrapint et al. (2015)).

**Figure 4.**
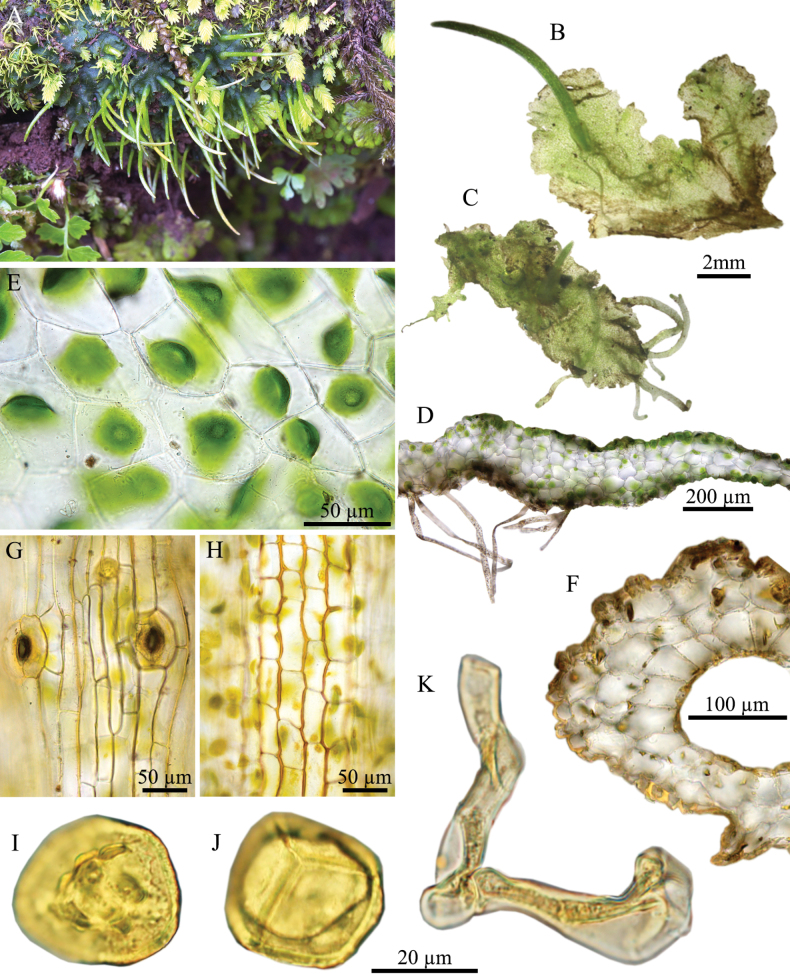
*Phaeoceros himalayensis* (Kashyap) Prosk. ex Bapna & G.G. Vyas. **A.** Plant in its natural habitat; **B, C.** Gametophytes and sporophytes; **D.** Cross section of thallus; **E.** Dorsal epidermal cells of thallus; **F.** Cross section of capsule wall; **G.** Epidermal cells of capsule and stoma; **H.** Innermost cells of capsule wall; **I.** Distal view of spore (LM); **J.** Proximal view of spore (LM); **K.** Pseudoelaters. Photographed by O. Suwanmala; based on *S. Chantanaorrapint & O. Suwanmala 3880A* (**A**), *3877* (**B–F, I–K**) and *4079* (**G, H**).

#### Description.

***Thallus*** bright green in fresh specimens, becoming yellowish green to brown when dry, growing prostrate, moderately adhering to substrate, forming irregular patches or sometimes fan-shaped colonies, irregularly branched into several lobes, thallus lobe lingulate to obovate, fan-shaped, or tapering toward apex, 4–12 mm long, 1–5 mm wide; margins nearly entire to irregularly crenulate, flat, rarely ascending upward; apex round to lobulate, frequently attenuated into tuber, rarely curving upward; tubers frequently present, occurring at apex, along margin, or on ventral surface of thallus, with a stalk to 5 mm long, ovoid to subspherical. ***Thallus in cross section*** plano-convex to concave-concave, 5–8 cells thick in the middle region. ***Dorsal epidermal cells*** irregular hexagonal to heptagonal, 42–100 × 25–48 µm. ***Chloroplasts*** 1 per cell, expanded, occupying nearly entire to about half of cell size, frequently contracted into round shape, pyrenoid smooth. ***Nostoc colonies*** scattered through the ventral side of thallus, appearing as dark dots. ***Rhizoids*** sparse, scattered on ventral surface, smooth in early stage, becomes pegged at maturity, hyaline to pale brown. ***Sexuality*** monoicous, weakly protandrous. Androecia sparse, slightly raised over the dorsal surface of thallus, (1–)2–4 antheridia per chamber. Archegonia embedded in thallus, connected to the upper surface, sparse, randomly scattered nearly thallus apex. ***Involucres*** erect, cylindrical, 1.1–2 mm long, mouth smooth to shallowly crenulate. ***Sporophytes*** frequent, capsules erect, cylindrical, up to 12 mm long at maturity; epidermal cells of capsule elongate-rectangular, 82–245 × 12–28 µm, thick-walled; stomata 57–83 × 40–53 µm, surrounded by 5–6 epidermal cells; assimilative layer 3–5 cells thick in cross section; the innermost cells of capsule elongate rectangular to hexagonal, 37–125 × 7–30 µm, pale brown to dark brown; columella consisting of 5–10 cells in cross section, brown to dark brown. ***Spores*** yellowish brown to dark brown, 27–35 µm in equatorial diameter; distal face with irregular verrucose projections, sometimes with aggregation in center; proximal face with distinct thin triradiate mark, finely vermiculate along its length; each facet finely vermiculate, with a central depression occasionally with sparsely surrounded by papillae. ***Pseudoelaters*** pale to dark brown at maturity, thin to thick-walled, occasionally branched, 1–3 cells long; pseudoelaters cells rectangular, without helicoidal band.

**Figure 5.**
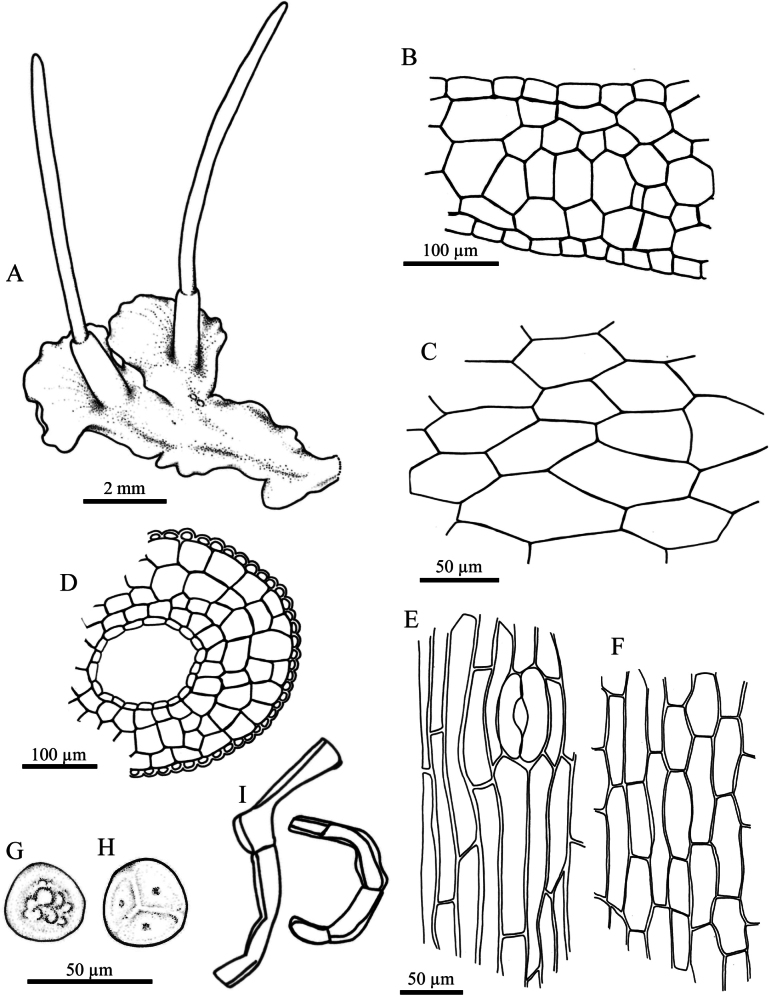
*Phaeoceros himalayensis* (Kashyap) Prosk. ex Bapna & G.G. Vyas. **A.** Gametophyte and sporophytes; **B.** Cross section of thallus; **C.** Dorsal epidermal cells of thallus; **D.** Cross section of capsule; **E.** Epidermal cells of capsule and stoma; **F.** Innermost cells of capsule wall; **G.** Distal view of spore; **H.** Proximal view of spore; **I.** Pseudoelaters. Drawn by O. Suwanmala; based on *S. Chantanaorrapint & O. Suwanmala 3877*.

#### Distribution, habitat, and ecology.

*Phaeoceros himalayensis* is currently known from India (Asthana and Srivastava 1991) and Thailand (Chantanaorrapint et al. 2015). It occurs on rocks and soil in open sites in grassland, pine-oak mixed montane deciduous, and subalpine forests at elevations between 1,000 and 2,200 m. It may grow associated with other bryophytes such as *Asterella khasyana* (Griff.) Pandé et al., *Cyathodium aureonitens* (Griff.) Mitt. and *Fissidens* spp.

#### Conservation status.

This species is not under immediate threat, due to many populations being found in India and northern Thailand, with the extent of occurrence (EOO) of over 900,000 km^2^ and its occurrence in protected areas. According to the IUCN criteria, the conservation status of *P. himalayensis* is classified as Least Concern (LC).

#### Specimens examined.

**India** • **Himachal Pradesh**: Shimla, Jakhu Tample, 2 Oct 2012, *Duckett et al. IW1* (QFA); • **Meghalaya**: West Khasi Hills, Thipringsong Forest, Nongstoin, ca 1,636 m elev., 15 Sep 2000, *A.P. Singh & M. Lal 208617* (LWG); • **Uttarakhand**: Almora, on way to Binsar, 1,969–2,272 m elev., 4 Oct 1991, *V. Nath & A.K. Asthana 205348C, 205359B* (LWG); Almora, on way to P. Nath, 2,500 m elev., 6 Oct 1991, *V. Nath & A.K. Asthana 205380* (LWG); • Mussoorie, Dehra Dun, Wood Stock College, 2,121 m elev., 3 Oct 1977, *S. Chandra 203378B* (LWG); • Dhanaulti, 2,121 m elev., 3 Oct 1977, *S. Chandra 203385* (LWG); • Nainital, on the way of Kilbury, 1,818 m elev., 12 Sep 2001, *A.P. Singh & V. Sahu 208943* (LWG); • Uttarkashi, Silkiara, 1,818 m elev., 15 Sep 1977, *S. Chandra 203229* (LWG); • **West Bengal**: Darjeeling, Himalayan Mountaineering Institute Road, ca 2,060 m elev., *A.K. Asthana & V. Sahu 224004* (LWG). **Thailand** • **Chiang Mai**: Chiang Dao, Angsalung base camp, 19°23'51.56"N, 098°53'21.08"E, 2,191 m elev., 11 Nov 2016, *S. Chantanaorrapint & O. Suwanmala 651* (PSU), 29 Aug 2017, *S. Chantanaorrapint & O. Suwanmala 2024* (PSU), 9 Oct 2019, *S. Chantanaorrapint & O. Suwanmala 3875, 3876, 3877* (PSU), 13 Nov 2020, *S. Chantanaorrapint & O. Suwanmala 4079, 4080* (PSU), 24 Nov 2022, *O. Suwanmala 854, 856, 857* (PSU); • Doi Luang Chiang Dao, 2,169 m elev., 19 Dec 2011, *S. Chantanaorrapint 2540* (PSU), 1 Nov 2013, *S. Chantanaorrapint & C. Promma 3123, 3126, 3215A* (PSU); • the trail to Kew Lom, 19°23'33.36"N, 098°53'24.40"E, 1,937 m elev., 12 Nov 2016, *S. Chantanaorrapint & O. Suwanmala 664, 695* (PSU), 28 Aug 2017, *S. Chantanaorrapint & O. Suwanmala 2006* (PSU), 9 Oct 2019, *S. Chantanaorrapint & O. Suwanmala 3880A* (PSU), 13 Nov 2020, *S. Chantanaorrapint & O. Suwanmala 4081, 4082* (PSU); • **Lam Phun**: Khun Tan National Park, 18°29'40.18"N, 099°17'17.45"E. 1,092 m elev., 20 Aug 2022, *S. Chantanaorrapint & O. Suwanmala 4474* (PSU); • **Tak**: Umphang, Thung Yai Naresuan, 19 Sep 2014, *S. Chantanaorrapint 2756* (PSU).

#### Taxonomic notes.

*Phaeoceros himalayensis* is characterized by 1) the presence of tubers at the ventral and apical regions of the thallus, 2) the distal face of the spore covered by irregular verrucose projections, 3) the presence of a central depression on each proximal face, and 4) yellowish brown to dark brown sporophytes at maturity. *Phaeoceros himalayensis* resembles *P. kashyapii* in having irregular verrucae on the distal face of the spore, but it differs from the latter by the presence of a central depression on each proximal face.

The sexual condition of *P. himalayensis* has been subject to different interpretations. Kashyap (1915) first described *Anthoceros himalayensis* (= *P. himalayensis*) as a dioicous plant with male and female thalli differing in size. However, Mehra and Handoo (1953) noted that it was monoicous but protandrous, based on their collections from Mussoorie, Simla, and Nainital in India. Proskauer (1967) and Asthana and Srivastava (1991) also stated that *P. himalayensis* was monoicous, while Chantanaorrapint et al. (2015) described it ambiguously as monoicous and dioicous. Based on specimens examined as part of this study, *P. himalayensis* is monoicous.

### ﻿ Phaeoceros kashyapii

Taxon classificationPlantaeNotothyladalesNotothyladaceae

A.K. Asthana & S.C. Srivast., Bryophyt. Biblioth. 42: 129, pl. 30, 48. 1991.

9B5A096F-A901-5750-A601-FD6A1D453272

Figs 6, 7, 11A, B

#### Type.

**India • Western Himalayas**: Deoban. 29 Sep 1976, *D.K. Singh & J.C.J. 2170/76* (holotype: LWU!).

**Figure 6.**
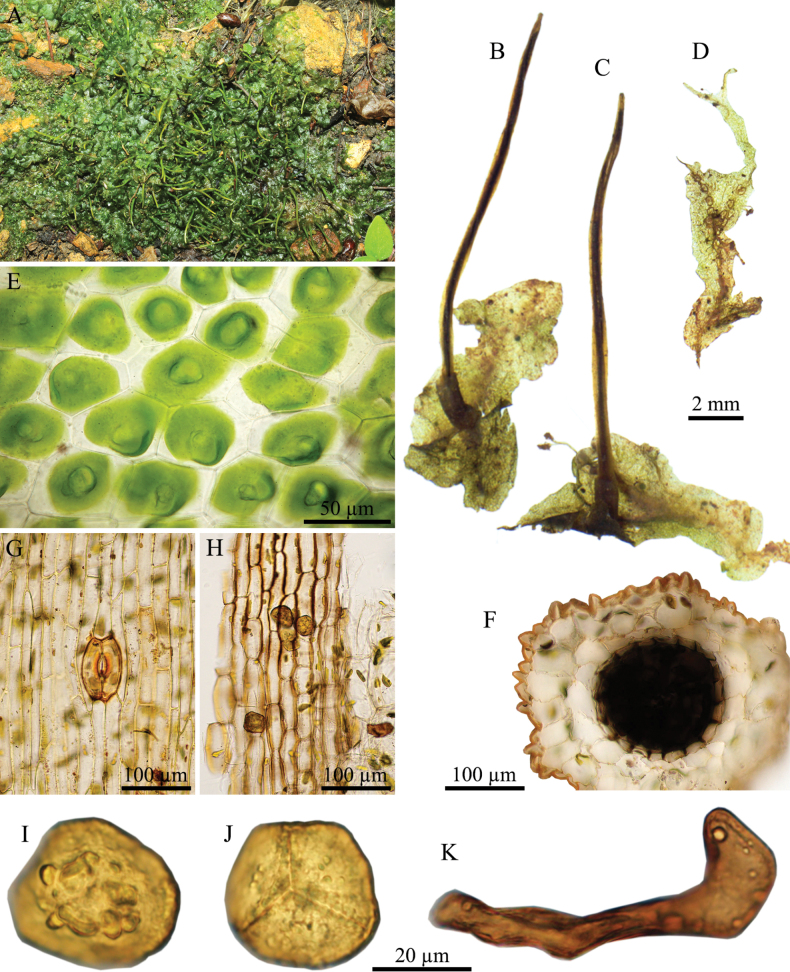
*Phaeoceros kashyapii* A.K. Asthana & S.C. Srivast. **A.** Plant in its natural habitat; **B, C.** Gametophytes and sporophytes; **D.** Dorsal view of thallus showing antheridium chamber; **E.** Dorsal epidermal cells of thallus; **F.** Cross section of capsule; **G.** Epidermal cells of capsule and stoma; **H.** Innermost cells of capsule wall; **I.** Distal view of spore (LM); **J.** Proximal view of spore (LM); **K.** Pseudoelater. Photographed by O. Suwanmala; based on *S. Chantanaorrapint & O. Suwanmala 3898* (**B–D**) and *3901* (**A, E–K**).

#### Description.

***Thallus*** bright green to yellowish green in fresh samples, become yellowish green to dark brown when dry, growing prostrate with moderately adhering to substrate, forming irregular patches or fan-shaped colonies, irregularly branched into several lobes, thallus lobe lingulate to obovate, or fan-shaped, the base usually narrower than the apex, 7–20 mm long, 2–6 mm wide; margins nearly entire to irregularly crenulate, sometimes lobulate along the margin, flat, rarely ascending upward; apex generally lobulate, broad, occasionally attenuate into apical tuber, rarely curving upward; tubers frequently present, occurring at apex, along margin, or on ventral surface of thallus, with a stalk to 5 mm long, ovoid to subspherical. ***Thallus in cross section*** plano-convex to concave-convex, 4–8 cells thick in the middle region. ***Dorsal epidermal cells*** irregular pentagonal to heptagonal, 30–150 × 20–55 µm. ***Chloroplasts*** 1 per cell, expanded, occupying nearly entire to half of cell size, frequently contracted into round shape, pyrenoid smooth. ***Nostoc colonies*** scattered ventrally, appearing as dark spots. ***Rhizoids*** sparse, scattered mainly along the middle region of ventral surface, smooth in early stage, becomes pegged at maturity, hyaline to pale brown. ***Sexuality*** monoicous or strong protandrous, androecia and archegonia not seen. ***Involucres*** erect, conical-cylindrical, 1.2–1.7 mm long, 2–4 cells thick, mouth smooth to shallowly crenulate. ***Sporophytes*** frequent, capsules erect, cylindrical, up to 15 mm long at maturity; epidermal cells of capsule elongate-rectangular, 117–300 × 10–28 µm; stomata 70–83 × 42–85 µm, surrounded by 5–6 epidermal cells; assimilative layer 2–4 cells thick in cross section; the innermost capsule cells elongate rectangular to hexagonal, 37–155 × 7–28 µm, pale brown to brown; columella consisting of 4–8 cells in cross section, brown to dark brown. ***Spores*** yellowish brown to dark brown, 30–38 µm in equatorial diameter; distal face with irregular large verrucose confined to the center; proximal face with distinct thin triradiate mark, finely vermiculate along its length; each facet finely vermiculate, frequently with sparse papillae confined to the center of each facet. ***Pseudoelaters*** thin to thick-walled, occasionally branched, 1–3 cells long; pseudoelaters cells irregular rectangular, yellowish brown to dark brown, without helicoidal band.

**Figure 7.**
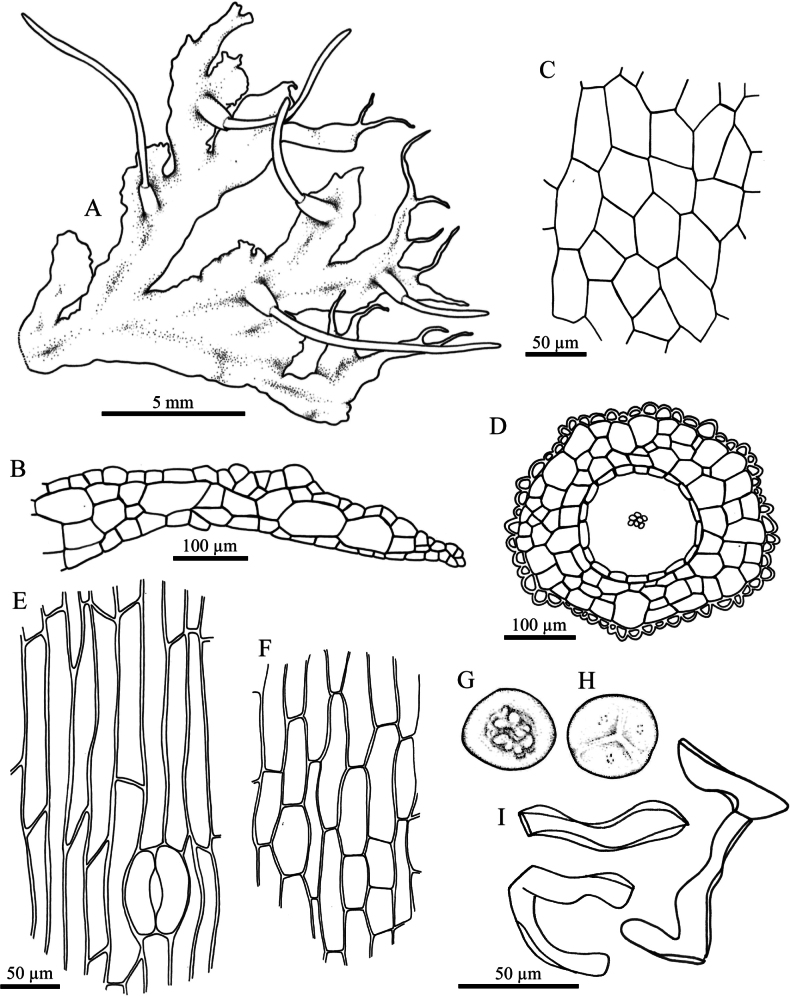
*Phaeoceros kashyapii* A.K. Asthana & S.C. Srivast. **A.** Gametophyte and sporophytes; **B.** Cross section of thallus; **C.** Dorsal epidermal cells of thallus; **D.** Cross section of capsule; **E.** Epidermal cells of capsule and stoma; **F.** Innermost cells of capsule wall; **G.** Distal view of spore; **H.** Proximal view of spore; **I.** Pseudoelaters. Drawn by O. Suwanmala; based on *S. Chantanaorrapint & O. Suwanmala 3901*.

#### Distribution, habitat, and ecology.

*Phaeoceros kashyapii* usually grows in open areas in pine-oak forests ranging from 900–2,200 m in elevation.

#### Distribution.

India and Thailand (Asthana and Srivastava 1991).

#### Conservation status.

*Phaeocero kashyapii* has an estimated EOO of 200,894 km^2^, suggesting a status of Least Concern (LC), while its area of occupancy (AOO) is 36 km^2^, which would place it in Endangered (EN). Indian populations are more widely distributed and can be found in disturbed areas, while Thai populations are rare and found alongside walking trails in the conservation areas. However, it is unclear whether the species continued occurrence at some of these sites. Its habitats are probably impacted by development and human disturbance. Therefore, *P. kashyapii* could be qualified as Endangered (EN) according to the IUCN Red List Criteria B2ab(iii) (IUCN 2024).

#### Specimens examined.

**India • Uttarakhand**: Mussoorie, Dehra Dun, Wood Stock College, 2,121 m elev., 3 Oct 1977, *S. Chandra 203383* (LWG); Nainital, on the way to Kilbury, ca 1,818 m elev., 12 Sep 2001, *A.P. Singh & V. Sahu 208947* (LWG); • Nainital, on the way to Tippin top, ca 2,181 m elev., 13 Sep 2001, *A.P. Singh & V. Sahu 208975A* (LWG); • Uttarkashi, Silkiara, 1,818 m elev., 15 Sep 1977, *S. Chandra 203222, 203225B, 203225C* (LWG); • Syana Chatti, Janki Chatti, 1,818 m elev., 20 Sep 1977, *S. Chandra 203253A* (LWG); • **Western Himalayas**: Deoban, 29 Sep 1976, *D.K. Singh & J.C. Joshi 2170/76* (LWU). **Thailand • Chiang Mai**: Chiang Dao, Doi Sam Phe Nong, ca 1,500 m elev., 10 Oct 2019, *S. Chantanaorrapint & O. Suwanmala 3898, 3900, 3901* (PSU); • Pang Woa, 19°24'33.05"N, 098°51'35.46"E, 1,178 m elev., 13 Nov 2016, *S. Chantanaorrapint & O. Suwanmala 718A* (PSU); • **Lum Phun**: Khun Tan National Park, 18°29'55.34"N, 099°16'43.92"E, 904 m elev., 11 Oct 2019, *S. Chantanaorrapint & O. Suwanmala 3920* (PSU).

#### Taxonomic notes.

*Phaeoceros kashyapii* is similar to *P. himalayensis* in several morphological characters of the gametophyte and sporophyte. Both are monoicous and usually grow in irregular patches. The thallus frequently produces tubers with long stalks on the ventral side, margins, and apex. Sporophytes are no longer than 15 mm, turning yellowish brown at maturity with an adhering valve tip. However, *P. kashyapii* differs from *P. himalayensis* by its spores lacking a central hollow on the proximal facet and usually bearing a small cluster of minute papillae restricted to the central region of each facet.

The examination of the holotype of *P. kashyapii* revealed that the spores of the type collection have a depression at the center of each facet, which morphologically resembles *P. himalayensis*. This indicates that the holotype of *P. kashyapii* is possibly mixed. The original publication of *P. kashyapii* also noted that it was found associated with *P. himalayensis*. Nevertheless, all specimens examined in this study display characteristics that belong to *P. kashyapii*, based on the first description and photographs provided by Asthana and Srivastava (1991). Based on collections from Thailand, gametophytes are strongly protandrous, with antheridia or antheridial chambers rarely found in sporophyte-bearing thalli. However, it is quite clear that the Indian population of *P. kashyapii* presents a monoicous plant producing male and female gametes on the same thallus.

The proximal spore architecture of *P. kashyapii* is typically finely vermiculate, with sparse minute papillae limited to the center of each facet. However, the papillae may be present in small numbers or occasionally absent. In such cases, it is quite difficult to distinguish spores of *P. himalayensis* and *P. kashyapii* using a light microscope. Therefore, careful investigation of the proximal face of the spore is required for accurate species recognition.

### ﻿ Phaeoceros stenothallus

Taxon classificationPlantaeNotothyladalesNotothyladaceae

Suwanmala & Chantanaorr. sp. nov.

B9EB41C8-3587-5E0B-A0CA-5B4B559800C2

Figs 8, 9, 11C–F

#### Type.

**Thailand • Chiang Mai**: Chiang Dao, Denya Khad, 1,413–1,500 m, 14 Nov 2020, *S. Chantanaorrapint & O. Suwanmala 4086* (holotype: PSU!, isotype: NICH!, QFA!).

**Figure 8.**
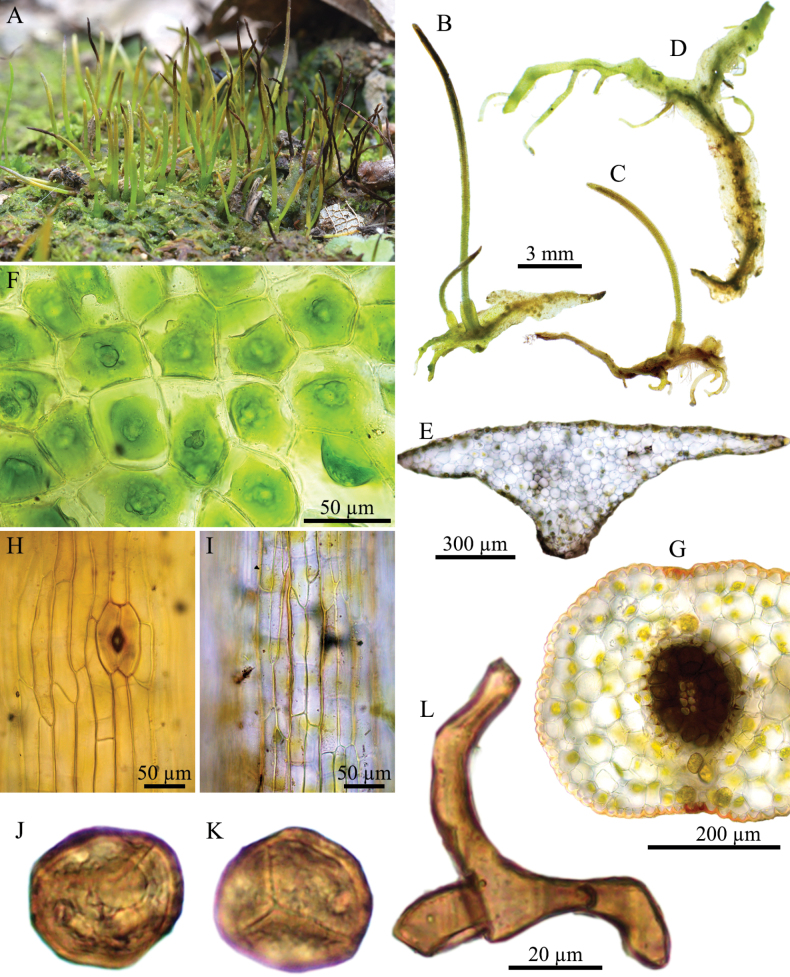
*Phaeoceros stenothallus* Suwanmala & Chantanaorr. **A.** Plant in its natural habitat; **B, C.** Female gametophytes and sporophytes; **D.** Male gametophyte with numerous tubers; **E.** Cross section of thallus; **F.** Dorsal epidermal cells of thallus; **G.** Cross section of capsule; **H.** Epidermal cells of capsule and stoma; **I.** Innermost cells of capsule wall; **J.** Distal view of spore (LM); **K.** Proximal view of spore (LM); **L.** Pseudoelater. Photographed by O. Suwanmala; based on *S. Chantanaorrapint & O. Suwanmala 3453* (**A**), *3845* (**F**), *4048* (**H, I**) and *4086* (**B–E, G, J–L**).

#### Diagnosis.

*Phaeoceros stenothallus* is similar to *P. himalayensis* and *P. kashyapii* but differs in dioicous sexuality, a narrow thallus never broader than 3 mm, wide and the vermiculate spore with hump-like projection on distal face.

**Figure 9.**
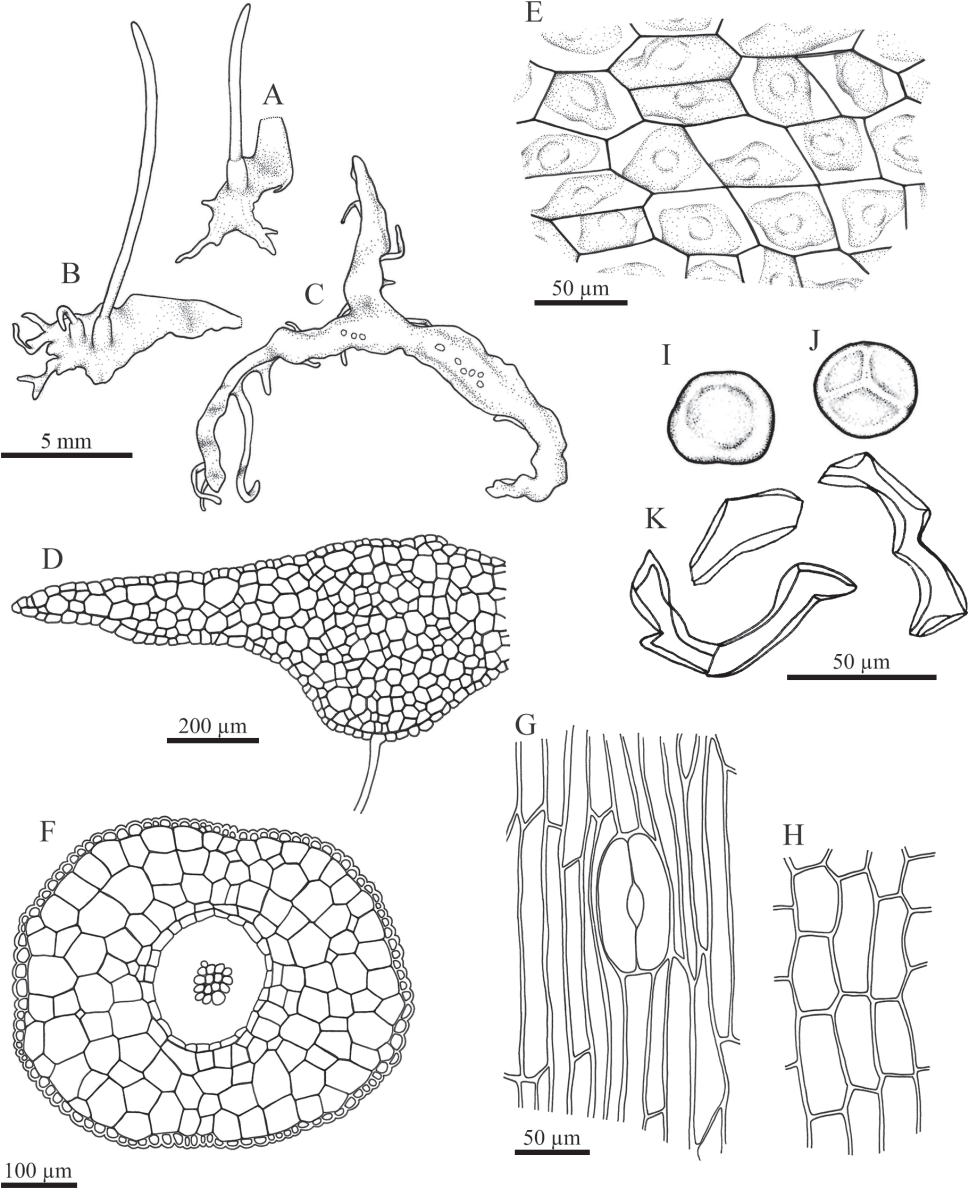
*Phaeoceros stenothallus* Suwanmala & Chantanaorr. **A, B.** Female thalli with apical tendrils and sporophytes; **C.** Male thallus with ventral tuber, showing antheridium chambers scattered on the dorsal surface; **D.** Cross section of thallus; **E.** Dorsal epidermal cells of thallus; **F.** Cross section of capsule; **G.** Epidermal cells of capsule and stoma; **H.** Innermost cells of capsule wall; **I.** Distal view of spore; **J.** Proximal view of spore; **K.** Pseudoelaters. Drawn by O. Suwanmala; based on *S. Chantanaorrapint & O. Suwanmala 4086*.

**Figure 10.**
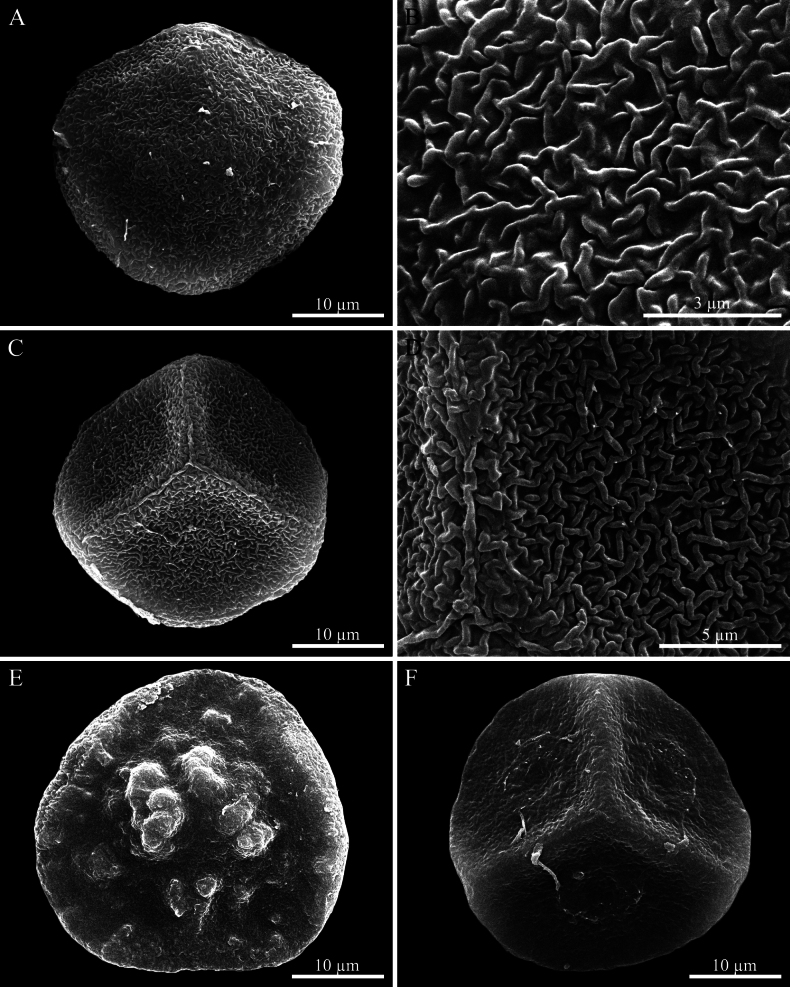
Scanning electron micrograph of spores. **A–D.***Phaeoceros aequatus*: **A.** Distal view of spore; **B.** Close-up of distal face; **C.** Proximal view of spore with a distinct triradiate mark; **D.** Close-up of proximal face showing trilete mark. **E, F.***P. himalayensis*: **E.** Distal view of spore; **F.** Proximal view of spore with a distinct triradiate mark. Photographed by O. Suwanmala; based on *S. Chantanaorrapint & O. Suwanmala 3877* (**E, F**) and *4070* (**A–D**).

**Figure 11.**
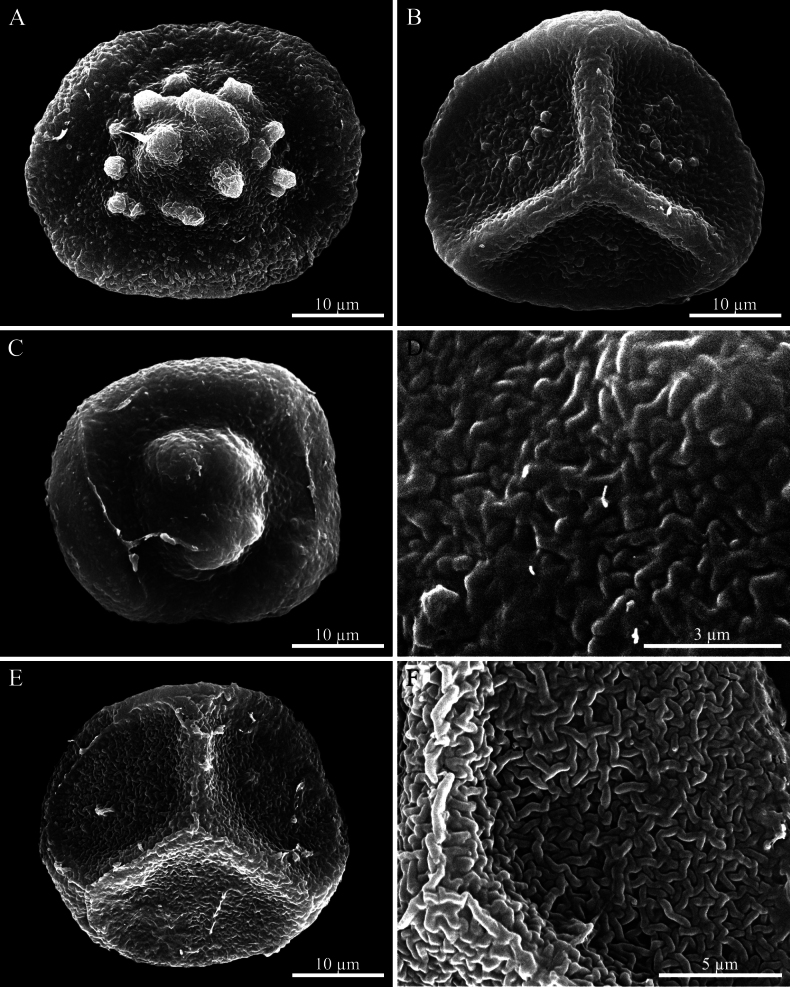
Scanning electron micrograph of spores. **A, B.***Phaeoceros kashyapii*: **A.** Distal view of spore; **B.** Proximal view of spore with a distinct triradiate mark. **C–F.***P. stenothallus*: **C.** Distal view of spore; **D.** Close-up of distal face; **E.** Proximal view of spore with a distinct triradiate mark; **F.** Close-up of proximal face showing trilete mark. Photographed by O. Suwanmala; based on *S. Chantanaorrapint & O. Suwanmala 3901* (**A, B**) and *4086* (**C–F**).

#### Description.

***Thallus*** bright to dark green in fresh material, becoming yellowish green to brown when dry, growing prostrate with loosely to moderately adhering to substrate, forming irregular patches or colonies, compactly, irregularly dichotomous branched into several lobes, thallus lobe strap-shaped, narrow, sometimes tapering toward apex, become boarder in sporophyte-bearing thalli, up to 12 mm long, 0.8–3 mm wide. margins entire to wavy, rarely crenulate, usually flat; apex usually attenuate, gradually tapering into apical tuber, sometimes shallowly lobulate; tubers always present, occurring at apex, along margins, and on the ventral surface, well-developed stalk, the tip with rounded end or ovoid to globose node, sometimes branched, up to 10 mm long. ***Thallus in cross section*** biconvex or plano-convex, with 6–16 cells thick in the middle region. ***Dorsal epidermal cells*** irregular pentagonal to heptagonal, 23–105 × 18–50 µm. ***Chloroplasts*** 1 per cell, occupying nearly entire to half of cell size, frequently contracted into round shape, occasionally folded at margin or star-like shape. ***Nostoc colonies*** irregularly distributed, sparse, appearing as dark spots. ***Rhizoids*** sparse to densely scattered along the middle region of ventral surface, hyaline to brown. ***Sexuality*** dioicous. Androecia abundant at the middle of thallus, distinctively raised over the dorsal surface of thallus, usually 2–3 antheridia per chamber; antheridia subglobose to globose, 2-tiered stalk with quadriseriate cells, 220–240 × 150–180 µm. Archegonia not seen. ***Involucres*** erect, conical-cylindrical, up to 2 mm long, 2–5 cells thick, mouth smooth to shallowly crenulate. ***Sporophytes*** often, capsules erect with slightly bending tip, cylindrical, 5–10(–12) mm long; epidermal cells of capsule elongate-rectangular, 80–207 × 10–31 µm, thick-walled; stomata 85–92 × 50–54 µm, surrounded by 6–7 cells; assimilative layer 3–5(–6) cells thick in cross section; the innermost capsule cells subquadrate to rectangular, 30–110 × 20–38 µm, brown to dark brown; columella consisting 8–16 cells in cross section, reddish brown to dark brown. ***Spores*** yellowish brown to dark brown, 29–38 µm in equatorial diameter; distal face hump-like projection without verrucose; proximal face with distinct thin triradiate mark; ornamentation finely vermiculate throughout the spore. ***Pseudoelaters*** thin to thick-walled, rarely branched, 1–2 celled; pseudoelaters cells rectangular, brown to dark brown, without helicoidal band.

#### Etymology.

The epithet “*stenothallus*” refers to the narrow thallus.

#### Habitat and ecology.

*Phaeoceros stenothallus* grows abundantly in open areas in mixed deciduous dipterocarp forest and pine-oak forest at elevations between 1,000 and 2,200 m in elevation.

#### Distribution.

Endemic to Thailand.

#### Conservation status.

*Phaeoceros stenothallus* has been found in the north to northwestern part of Thailand, with abundant populations in Chiang Dao Wildlife Sanctuary and spare populations in Mon Long, Doi-Suthep Pui National Park, and Umphang Wildlife Sanctuary. The EOO is estimated to be about 4900 km^2^ and the AOO is 36 km^2^. The majority of its habitats are unique, occurring in moist but seasonally dry forests, typically on limestone bedrock areas. The species has a risk of habitat disturbance due to human activities and grazing, which may degrade the quality and stability of its natural environment. Therefore, *P. stenothallus* is here suggested to be Endangered according to IUCN Red List criteria B1ab(iii)+B2ab(iii) (IUCN 2024).

#### Specimens examined.

**Thailand • Chiang Mai**: Chiang Dao, Doi Sam Phi Nong, 10 Oct 2019, *S. Chantanaorrapint & O. Suwanmala 3895, 3896* (PSU); • Denya Khad station to Dong Noi, 19°22'35.38"N, 098°50'06.41"E, 1,413–1,500 m elev., 6 Sep 2012, *S. Chantanaorrapint, J. Inuthai & C. Promma 1573* (PSU), 10 Nov 2016, *S. Chantanaorrapint & O. Suwanmala 604, 605* (PSU), 10 Oct 2019, *S. Chantanaorrapint & O. Suwanmala 3902, 3904, 3907A* (PSU), 14 Nov 2020, *S. Chantanaorrapint & O. Suwanmala 4083, 4084, 4086* (PSU); • Muang Kong - Wiang Haeng boarder, 19°28'N, 098°41'E, 814–825 m elev., 29 Oct 2018, *S. Chantanaorrapint & O. Suwanmala 3453, 3456* (PSU); 6 Oct 2019, *S. Chantanaorrapint & O. Suwanmala 3845, 3846* (PSU); • Na Lao Village, 16 Oct 2020, *S. Chantanaorrapint & O. Suwanmala 4073, 4075* (PSU), 3 Oct 2021, *S. Chantanaorrapint & O. Suwanmala 4098* (PSU), 19°24'42.47"N, 098°49'57.33"E, 1,089 m elev., 7 Oct 2019, S. *Chantanaorrapint & O. Suwanmala 3850, 3853* (PSU); • the road to Chiang Dao trail, 19°19'55.95"N, 098°54'32.65"E, 807 m elev., 7 Oct 2019, *S. Chantanaorrapint & O. Suwanmala 3855, 3860, 3861* (PSU); • Mae Rim, Mon Long, 18°55'11.27"N, 098°50'25.00"E, 1,350 m elev., 4 Oct 2021, *S. Chantanaorrapint & O. Suwanmala 4104, 4106* (PSU); • **Tak**: Umphang, Doi Mamuang Sam Muen, 15°52'03.44"N, 098°37'09.80"E, 1,119 m elev., 26 Sep 2020, *S. Chantanaorrapint & O. Suwanmala 4048, 4054* (PSU).

#### Taxonomic notes.

*Phaeoceros stenothallus* can be distinguished by a combination of the following characters: 1) dioicous sexuality, 2) a narrow thallus never broader than 3 mm wide, 3) the presence of long-stalked tubers on the ventral side, margins, and apex of the thallus, 4) the yellowish brown to dark brown sporophyte at maturity with an adhering valve tip, and 5) the vermiculate spore with a hump-like projection on the distal face.

*Phaeoceros stenothallus* may be confused with *P. himalayensis* and *P. kashyapii* when they are sterile, with a linear and forked thallus. However, *P. stenothallus* differs from both species by its dioicous sexual condition and the absence of verrucae on the distal face of the spores. Furthermore, this species resembles members of the genus *Phymatoceros* (especially *P. bulbiculosus* (Brot.) Stotler et al. and *P. phymatodes* (M. Howe) R.J. Duff et al.) in general appearance, such as its narrow thallus, long-stalked tubers, dioicous gametophytes, and spore morphology. However, members of *Phymatoceros* differ from the new species by their larger spores, thicker-walled epidermal cells of the capsule, and tubers arising from apical cells.

## Supplementary Material

XML Treatment for Phaeoceros

XML Treatment for Phaeoceros

XML Treatment for Himalayanus

XML Treatment for Phaeoceros aequatus

XML Treatment for Phaeoceros himalayensis

XML Treatment for Phaeoceros kashyapii

XML Treatment for Phaeoceros stenothallus

## ﻿Additional information

### Use of AI

No use of AI was reported.

### Author ORCIDs

Orawanya Suwanmala https://orcid.org/0000-0002-1113-7614

Juan Carlos Villarreal A. https://orcid.org/0000-0002-0770-1446

Sahut Chantanaorrapint https://orcid.org/0000-0002-9739-0994

### Data availability

All of the data that support the findings of this study are available in the main text or Supplementary Information.

## Supplementary materials

Supplementary material 1 List of specimens used in the phylogeny analysis Data type xlsx Explanation note **table S1.** List of specimens used in the phylogeny analysis, including voucher information, GenBank accession numbers or the published sequences name together with original sources. newly generated sequences are bold. https://binary.pensoft.net/file/1490245This dataset is made available under the Open Database License (http://opendatacommons.org/licenses/odbl/1.0/). The Open Database License (ODbL) is a license agreement intended to allow users to freely share, modify, and use this Dataset while maintaining this same freedom for others, provided that the original source and author(s) are credited.Orawanya Suwanmala, Juan Carlos Villarreal A., Sahut Chantanaorrapint
